# “Did You Call Me?” 5-Month-Old Infants Own Name Guides Their Attention

**DOI:** 10.1371/journal.pone.0014208

**Published:** 2010-12-03

**Authors:** Eugenio Parise, Angela D. Friederici, Tricia Striano

**Affiliations:** 1 Max Planck Institute for Human Cognitive and Brain Sciences, Leipzig, Germany; 2 Cognitive Development Center, Central European University, Budapest, Hungary; 3 Department of Psychology, Hunter College, New York, New York, United States of America; University of Barcelona, Spain

## Abstract

An infant's own name is a unique social cue. Infants are sensitive to their own name by 4 months of age, but whether they use their names as a social cue is unknown. Electroencephalogram (EEG) was measured as infants heard their own name or stranger's names and while looking at novel objects. Event related brain potentials (ERPs) in response to names revealed that infants differentiate their own name from stranger names from the first phoneme. The amplitude of the ERPs to objects indicated that infants attended more to objects after hearing their own names compared to another name. Thus, by 5 months of age infants not only detect their name, but also use it as a social cue to guide their attention to events and objects in the world.

## Introduction

Infants are highly sensitive to the communicative social cues that others offer [1], [2]. Most infants experience social signals such as eye contact and smiling. Direct eye contact modulates infants' cognitive processes such as face [3], [4] emotion [5], [6], and object processing [7], [8]. For a review see [9].

Infants use others' social cues to guide their attention to the world. They show enhanced attention to objects that have been cued by joint attention cues such as eye contact and positive facial expressions [2]. In event related potential (ERP) studies, infants show an enhanced Negative central (Nc) component to objects cued by joint attention [7], [8]. The Nc is a well-known component related to infant recognition memory [10], [11] and enhanced cognitive attentional processing [12], [13]. ERP waveforms following the Nc may be involved in maintaining the information over a period of time. They are related to novelty detection [11], [14] and to attention [12], [15]. For a review see [16]. Infants increase attention when objects are cued by eye contact or joint attention [5], [17], [18]. However, the question remains whether other social signals are detected and used by young infants when processing the world.

Communicative cues like eye gaze are equal for all infants. But there is one communicative cue that is unique to each individual infant: the infant's own name. Infants' sensitivity to their own first name has only been moderately investigated. Infants listen longer to their own names compared to other names by 4.5 months of age, as demonstrated by the head-turning technique [19]. Infants also respond differently to a close approximation of their own names. If a name differing only in the first phoneme from the infant's own name is heard, infants show no listening preference [20]. Moreover, 6- but not 4.5-month-olds preferentially respond to the word “baby” but do not show this effect for the word “mommy”. This suggests that infants listen preferentially to words typically directed to themselves, such as their own names and “baby”.

Research has focused on the role of the infant's own name in early language development. It has been hypothesized that infants use their own name to identify the next word in the speech stream. Available data are inconclusive. Mandel-Emer and Jusczyk [20] failed to provide data supporting this claim. However, they found that 6-month-olds preferentially listen to sentences containing their own name, compared to sentences containing strangers' names. Bortfeld, Morgan, Golinkoff and Rathbun [21] found that 6-month-olds prefer words that, in previously familiarized sentences, were preceded by their own name. This ability was present for the word “mommy” as well, but not for the word “Tommy”, suggesting that infants use the first phoneme to differentiate between the two words. Differences in experimental procedures may explain these contradictory results.

A stable and detailed representation of one's own name plays a role in language acquisition, but might also be important in social interaction. Neuroscience research in adults suggests that the own name is special. Using a passive listening oddball paradigm, Folmer and Yingling [22] found an auditory P3 component only in response to the subject's own name compared to other first names. When uttered by a familiar voice, an own name elicits more robust ERP responses of involuntary attention switching (a P3, but also a Mismatch negativity (MMN), respectively related to target recognition and automatic pre-attentive detection to changes in repetitive stimulation) and a large late slow wave at parietal sites [23] (this slow wave is taken to reflect brain activity contributing to the retrieval of information for accurate recognition judgments). These adults' data show that the own name is an attention-grabbing stimulus at early (MMN) and middle/late stage of the stimulus processing (P3 and slow wave). Although infants' ERP components do not always map onto adults' components, these results encourage the use of ERPs as a sensitive measure of the infants' brain response to the own name. For preverbal infants at age of 5 months, differences might be expected in ERP responses related to phonological processing, such mismatch effects, expressed as early positivities, and/or as middle latency negativities in the infants' ERP. For a review see [24].

In adults, neuroimaging research shows that brain areas active during own name listening include the medial prefrontal cortex, temporal poles, superior temporal cortex near the temporoparietal junction and the precuneus [25], [26]. These areas are also involved in self-recognition and mentalizing, the ability to attribute mental states to self and others. Interestingly, Kampe, Frith and Frith [27] found that hearing one's own name vs. a stranger's name and watching pictures of faces displaying mutual vs. averted eye gaze results in overlapping brain activation, specifically the right medial prefrontal cortex and the left temporal pole. A recent optical brain imaging study [28] using a similar paradigm in 5-month-old infants showed that young infants also recruit prefrontal regions when processing communicative signals of different modalities, although not directly overlapping. The data indicate that young infants selectively processed and attended to ostensive communicative signals directed to the self.

For human infants, name cues may be especially important. Infants may also rely on vocal social cues more than visual cues. Infants respond to the voice from early months [29], [30] and guide their behavior based on vocal cues during social referencing [31], [32].

Thus the own name appears to be of particular importance to the humans.

**Figure 1:**
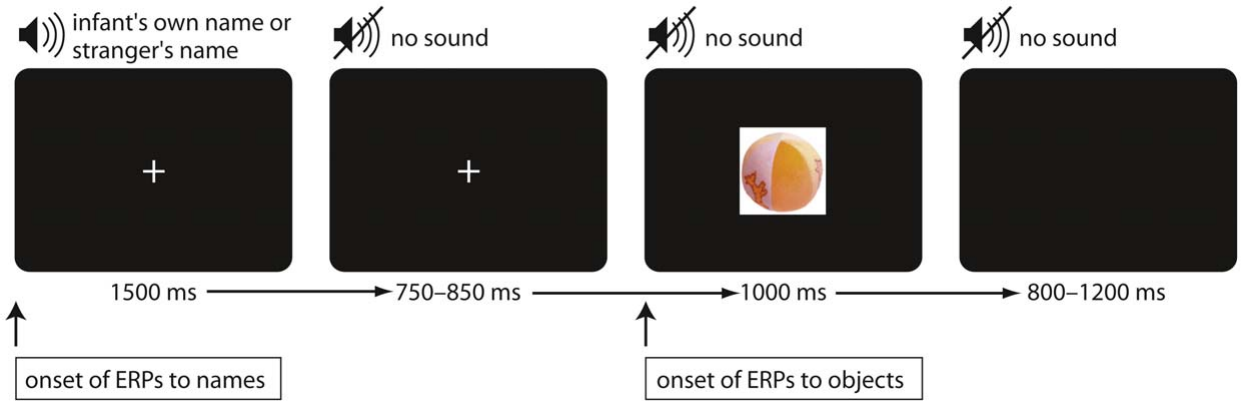
Example of experimental trial. Infants heard their own name or a stranger's name in a time window of 1500 ms. After a random interval they saw the picture of a toy for 1000 ms. This trial led to the two ERP averages (to names and to objects) for each participant.

In the current study we assessed whether listening to their own name directs infants' attention to objects. Using ERP methodology, we investigated how 5-month-old infants process their own names, which neural correlates are involved, and how the own name enhances infants' attention to objects (see Figure 1). We tested two groups of 5-month-olds: one group heard ten different control names and the other group heard one control name. Compared to previous behavioral studies [19], [20], this design allowed us to rule out the possibility that infants react to their own name because it was the only constant sound during stimuli presentation. In addition, this experimental design allowed us to study what phonological cues infants use to process their own name compared to control stimuli that were either constant or variable. We predicted that infants would differentiate their own name from a stranger's name regardless of the experimental condition. We also predicted that infants would differentiate their names from other names from the first phoneme if phonologically allowed [21], and that this would be reflected in an early ERP effect to the own name vs. control stimuli. Given the lack of ERP studies on word processing with very young infants, we tried to formulate an *a priori* prediction on specific auditory components of name processing. Based on the available data [33], we expected to observe an early positive deflection that was higher in amplitude when infants heard their own name compared to a stranger's name. Based on the ERP literature for words processing in older infants [33], [34], [35], we also expected a middle-latency negativity effect for the own name compared to the stranger's name.

By showing infants an object after each name, we focused on the way they processed new objects as a function of their own name. If the infant's own name acted like an ostensive cue [36], we predicted that components like the Nc, and perhaps event related potentials following the Nc (electrophysiological markers of attention in infants) would be enhanced to objects that have been cued by the infants' own name.

## Results

### ERPs to names

**Figure 2:**
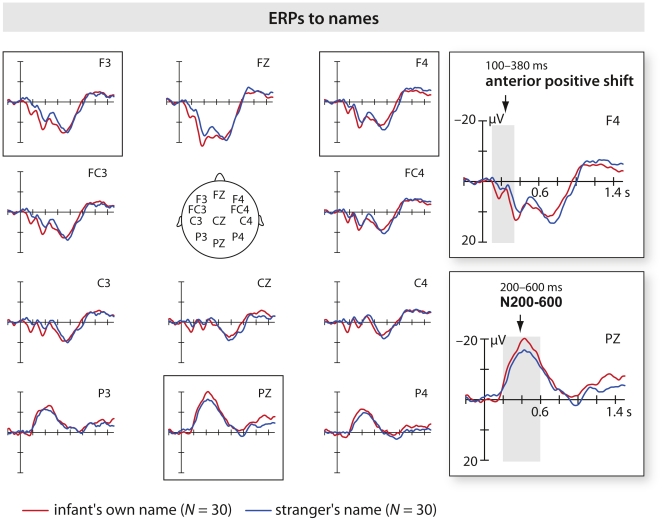
ERPs in response to names across groups. Auditory grand average collapsed over groups on frontal, central and parietal channels. Arrows highlight analyzed components. The grey bar indicates the time interval of averaged waves. The horizontal tick mark, 0.2 s; vertical tick mark, 10 µV. Negative is plotted up. The infant's own name is higher in amplitude on the early anterior positive shift and on the N200-600 component.

**Figure 3:**
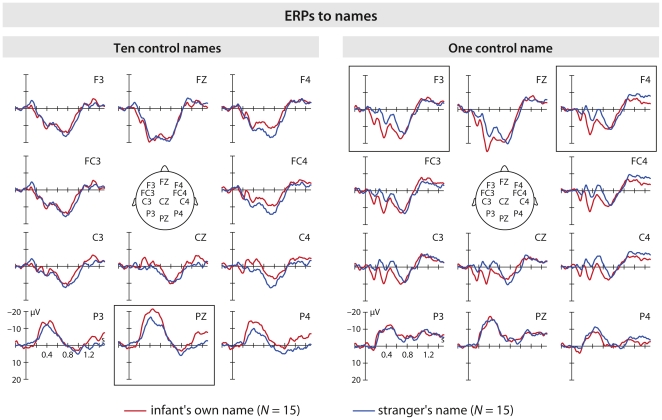
ERPs in response to names separate by groups. Auditory grand average split into the two groups on frontal, central and parietal channels. Horizontal tick mark, 0.2 s; vertical tick mark, 10 µV. Negative is plotted up. The infant's own name is higher in amplitude in the group with one control name on the anterior positive shift and in the group with ten control names on the N200-600 component.

ERPs to names are shown in Figure 2. The data in Figure 2 are collapsed across groups. In Figure 3 the data are presented for separate groups. To assess the topography and the time course of the auditory ERPs, two regions of interest (ROIs) as well as two time windows were chosen based on visual inspection of the grand average. First, we assessed ERPs on fronto-central channels between 100 and 380 ms after stimulus onset. ERPs were evaluated by averaging three electrodes in each hemisphere: left (F3, FC3, C3) and right (F4, FC4, C4). Second, we analyzed parietal channels between 200 and 600 ms to capture a possible middle-latency ERP effect. Both amplitude and latency were evaluated by averaging the peaks of parietal electrodes (P3, Pz, P4). ERPs were analyzed by a 2×2×2 ANOVA on fronto-central regions with Group (ten vs. one control name) as between-subjects factor, Name (infant's own name vs. stranger's name) and Hemisphere (left vs. right) as within-subjects factors. On parietal regions a 2×2 ANOVA was performed with Group as the between- and Name as the within-subjects factors, respectively. Scheffé and t-test were used for post hoc comparisons. Wilcoxon's test was used for non-parametric statistics.

As expected, the infant's own name showed more positive potential than the stranger's name maximal over anterior sites (see Figure 2 and 3). For this anterior positive shift, the ERP to the infant's own name was higher in amplitude than the ERP to the stranger's name (*F*_(1,28)_ = 5.23, *P*<.03, η_p_^2^ = .157). An interaction of Group by Name (*F*_(1,28)_ = 5.26, *P*<.03, η_p_^2^ = .158) and post hoc tests revealed that in the group with one control name the ERP to the infant's own name was higher in amplitude than the ERP to the stranger's name (*P*<.03), whereas this was not the case in the group with ten control names. Non-parametric statistics revealed that 12 out of 15 infants in the group with one control name showed the effect (*Z* = −2.33, *P*<.03). In the group with ten control names only 6 out of 15 showed the effect (*Z* = −.57, *P* = .57).

An N200-600 component on parietal channels also showed an interaction of Group by Name (*F*_(1,28)_ = 4.14, *P* = .051, η_p_^2^ = .129). A t-test within each group showed that the amplitude of the ERP to the infant's own name was more negative than the amplitude of the ERP to strangers' name (*t*(14) = −3.09, *P*<.008) in the ten control names group. This was not the case in the group that heard one control name (*t*(14) = .27, *P* = .79). Non-parametric tests showed that in the group with ten control names, 13 out of 15 infants showed the effect (*Z* = −2.56, *P*<.02), whereas in the group with one control name, only 7 infants showed the effect (*Z* = −.17, *P* = .87). No significant main effects or interactions were found for the N200-600 peak latency.

The anterior positive shift reported above started very early after sound onset. To rule out the possibility that this result was driven by a poor signal to noise ratio, due to the relatively small amount of averaged trials, the sample was split into two subgroups of 15 subjects each: infants with a number of trials below the median (<28.5) and infants with number of trials equal or above the median (≥28.5). A t-test on the average of all the electrodes considered in the anterior positive shift analysis revealed no difference between groups (*t*(28) = .39, *P* = .70).

To rule out the possibility that the observed ERP pattern was due to fast learning, we conducted additional analyses for the anterior positive shift and the N200-600 for the first and second half of the experiment (see Supplementary Information S1). Given that no significant interaction with the factor Split-Half was found, the observed pattern does not seem to support a fast learning hypothesis.

### ERPs to objects

**Figure 4:**
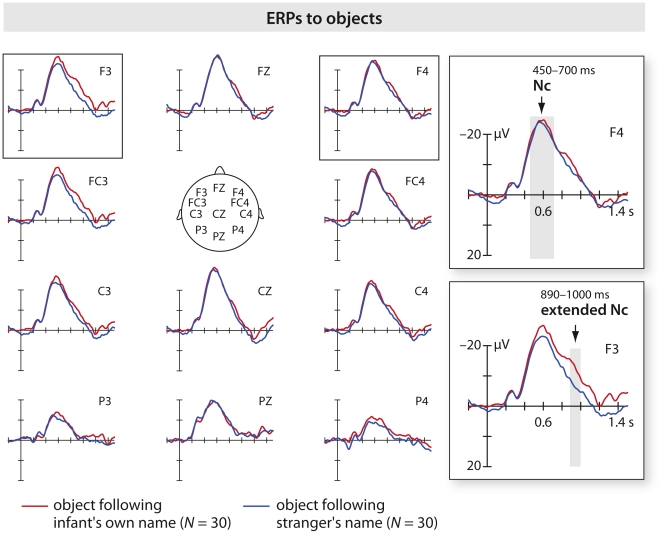
ERPs in response to objects across groups. Visual grand average collapsed over groups on frontal, central and parietal channels. Arrows highlight analyzed components. The grey bar indicates the time interval of averaged waves. Horizontal tick mark, 0.2 s; vertical tick mark, 10 µV. Negative is plotted up. Objects following the infant own name have a later Nc peak and a higher amplitude following the Nc.

Based on visual inspection of the grand average shown in Figure 4, two ROIs (left (F3, FC3, C3) and right (F4, FC4, C4)) as well as two time windows (450–700 ms and 890–1000 ms) were chosen to assess ERP effects to visually presented objects on fronto-central channels. Both 450–700 ms (peak amplitude and latency) and 890–1000 ms (average amplitude) ERPs were analyzed by a 2×2×2 ANOVA with Group, Name, and Hemisphere as factors.

**Figure 5:**
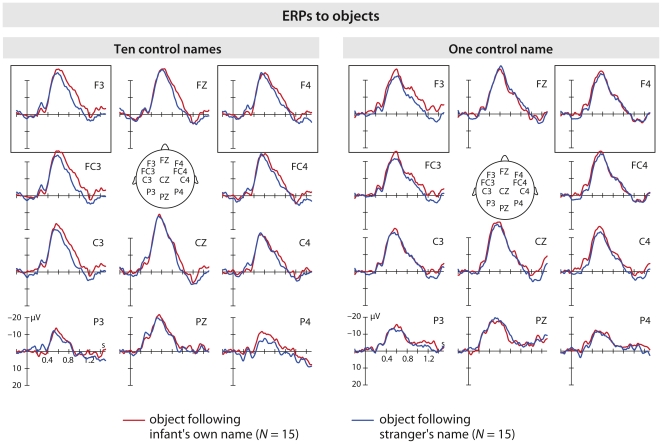
ERPs in response to objects separate by groups. Visual grand average split into the two groups on frontal, central and parietal channels. Horizontal tick mark, 0.2 s; vertical tick mark, 10 µV. Negative is plotted up. No group interactions were found for the visual components.

In the time window 450–700 ms the Nc latency to objects preceded by stranger's name peaked earlier than the Nc latency to objects preceded by infant's own name (*F*_(1,28)_ = 10.79, *P*<.003, η_p_^2^ = .278). Non-parametric statistics revealed that 21 out of 30 infants showed the effect (*Z* = −2.84, *P*<.005), among these infants, 10 belonged to the ten control names group and 11 to the one control name group. No significant effects or interactions were found for the Nc amplitude. As predicted, the ERP to objects preceded by the infant's own name had a higher amplitude than the ERP to objects preceded by stranger's name. This was true for the later time window 890–1000 ms in which an extended Nc was observed (*F*_(1,28)_ = 4.27, *P*<.05, η_p_^2^ = .132). Twenty out of 30 infants showed this effect (*Z* = −1.92, *P* = .05), among these infants, 11 belonged to the ten control names group and 9 to the one control name group. Additionally, a comparison of the ERP to objects across hemispheres revealed that the extended Nc to the own name condition on the left was higher in amplitude than the extended Nc to both conditions on the right (P<.002 and P<.003 respectively; interaction of Name by Hemisphere approaching significance *F*_(1,28)_ = 4.00, *P* = .055, η_p_^2^ = .125). No significant interactions with Group were found. Figure 5 shows ERPs to objects for each group separately.

## Discussion

We show that at 5 months of age infants use their own names as a social cue to process visually presented objects. ERPs to the infant's own name and its effect on object processing will be discussed in turn.

### Processing infant's own name

For name processing two effects were found: an early anterior positive shift and an N200-600 effect. Auditory ERPs demonstrated that infants are sensitive to the sound pattern of their own name early during processing, as indicated by the main effect of Name on the early anterior positive shift. This ERP pattern matches the one observed in young infants when hearing single words [33]. The differentiation process between the own and the stranger's name occurs very shortly after the stimulus onset (100–380 ms). This result, see also [21], suggests that infants differentiate their own name from a stranger's name starting from the first phoneme when this first phoneme differs from the control (stranger's) names. In 3- to 4-month-old infants, familiar words elicit a more pronounced early positivity on parietal sites than unfamiliar words [33]. This early positivity observed among infants at 3–4 months for both familiar and unfamiliar words, significantly larger (more positive) for familiar words, suggests an immature brain response. This result closely resembles the pattern we have observed over the fronto-central area, suggesting that the two ERP components may be of the same type. Specifically, in our study the anterior positive shift was most prominent in the group with one control name, suggesting that acoustic-phonological discrimination was more likely in this condition. The higher amplitude in the waveform, independently from its polarity, suggests that a larger population of neurons may have been active. Analogously, larger positive mismatch ERP has been observed to non-native rhythmic stress pattern compared to native stress pattern in German and French 4-month-old infants [37]. This positive ERP response is thought to be the infant analog of the adult MMN and to reflect additional effort in the perceptual processing of a stimulus that is deviant in the experimental setting and deviant in the group's native language. In the present study, the enhanced positivity might be interpreted as “phonological interest” to the own name. It is possible that early phonological discriminations are reflected in the positive MM response and in the anterior positive shift.

The N200-600 effect was evident as a more negative peak for the infant's own name compared to the stranger's name, and only significant in the group with ten different control names. This result was not unexpected. Starting at the age of 11 months, previous research [33], [34], [35] has shown a negativity between 200–400 ms for familiar compared to unfamiliar words. The present data suggest that such an effect can be observed at an early age. In particular, the result is for the infant's own name which should be a most familiar. However, a possible interpretation might be that negativities arise in infants younger than 11 months when there is repetition in variable ongoing stimulation [38]. If this were the case and the frequency of the repetition were the main factor driving the N200-600 effect, then a difference between groups should be observed as the infant's own name was repeated 50% of the trials in both groups (*t*(28) = −1.25, *P* = .22), but the control name was repeated 50% of the trials in the one name group, whereas each given control name was repeated 5% of the trials in the ten names group (*t*(28) = .59, *P* = .56). The post hoc t-test does not support this hypothesis. We have tried to exclude the possibility that the N200-600 effect is a result of the difference in the variability of the control name in the two groups. Nevertheless, such a possibility cannot be completely ruled out in the present study. Thus our results have to be taken with caution. We interpret our results as the likely reflection of more cognitive resources activated when the own name is presented in the context of ten other names compared to a single stranger's name.

Classically, a middle-latency negative deflection at centro-parietal channels is known as N400. The N400 is considered to be an electrophysiological marker of lexical-semantic processes [39] in adults and in older infants. It is taken to reflect the effort of integrating a stimulus into a semantic context [40]. However, N400 effects have also been reported for lexical-phonological processing of pseudowords showing more negative going waveforms than for non-words both in infants [34], [41] and adults [42], [43]. It is, however, unlikely that the centro-parietal negativity to own name observed here represented a lexical-semantic integration difficulty for 5-month-old infants. We propose that the N200-600 is functionally different from the classic N400 observed in adults and older infants and may reflect infants' allocation of increased neural resources, when processing their own name in the group of ten control names. Recent research with 6- and 12-month-olds is in line with this interpretation [44]. When comparing the processing of prosodically marked familiarized vs. unfamiliarized words 6-month-olds show an anterior positivity, whereas 12-month-olds show a fronto-central “N400”. These data (see also [33]) could mean that the anterior positive shift and the N200-600 are two sides of the same coin, with the anterior positive shift being the less mature ERP response and the N200-600 being the more mature ERP response. The more mature response may become obvious only in the ten names condition in which the infant's own name is more easily detected in the speech stream than in the more monotonous one name condition. Note, however, that no direct relation has been demonstrated here between the anterior positive shift and the N200-600.

Taken together, the present results confirm that infants are sensitive to the sound pattern of their own name, likely detecting it from the first phoneme. In addition the variability of control names influences the quality of auditory detection processes among young infants. The exact nature of these processes requires further investigation. Testing infants at different ages may be one way to understand the nature, emergence and development of the N200-600 [33], [44].

### Processing objects after hearing one's own name

Here we show that infants used their own name to direct their attention to objects. For object processing, an Nc peak was found earlier for objects following the strangers' names than the infant's own name. One possibility is that an object preceded by the own name was processed more slowly (the Nc reaches its lowest peak later) but deeper, requiring more neural resources, than an object preceded by the strangers' name. This hypothesis might also explain why we found an amplitude difference (890–1000 ms) following the Nc, which was larger for objects preceded by the infant's own name than for objects preceded by another name. In a study with 4-month-old infants, Hoehl and colleagues [45] found that the Nc to simultaneously presented gaze cue and object peaked earlier for the non-communicative condition (i.e. eye gaze averted from the object), but the event related potential following the Nc was higher for the communicative condition (i.e. eye gaze toward the object). We propose that more neural resources were allocated to process the objects preceded by the infant's own name with the own name acting as an attentional cue. From this perspective, our results are consistent with earlier work concerning eye gaze and object processing [7], [8], [45]. Together with the present work these studies suggest that young infants might perceive both their own name and eye gaze like communication starters, similar to adults [27]. Recent neuroimaging work by Grossmann, Parise and Friederici [28] seems to confirm this possibility. An alternative interpretation could be that the higher amplitude following the Nc indicates a larger effort for processing objects after hearing one's own name as a result of integrating the own name with the visually perceived objects. We hypothesize that by 5 months of age the own name is stored in the infant's memory. Hearing her own name prepares the infant to receive new relevant information. The observed higher potential following the Nc can be either interpreted as enhanced attention for the incoming new information - in this case new objects, or it may index increased integration effort. Increase effort is needed to integrate one's own name coming out from loudspeakers with the new visual information, namely the object appearing on the screen. The process may be modulated by the experience of a 5-month-old for whom the own name usually comes from a live person, who ostensively addresses the infant when referring to an object present in the room, and not from a loudspeaker. Future research is needed to address these alternatives in details, but the similarities of the present visual ERPs with those reported by other studies [7], [8] using live ERP paradigms (see next paragraph) suggest that the attentional hypothesis might be the most valid.

The second significant difference in ERPs to the objects occurs between 890–1000 ms, prominent over the left side of the scalp. This scalp distribution resembles that reported in papers using live joint attention ERP paradigms with infants [7], [8] in which the Nc showed differences over the left side of the scalp or over the midline. In these studies, both 5- and 9-month-olds paid more attention to the objects in the full joint attention situation (i.e. when the experimenter was looking to both the infant and the object). In infants, the neural network recruited by joint attention situations largely overlaps the adult brain network and involves the left dorsal prefrontal cortex [46]. It is also notable that the shape of the ERPs to objects in this study is extremely close to that reported by Striano, Reid et al. [8] with 9-month-olds in their study of object processing in joint attention situation. By commenting this result in a review paper, Grossmann and Johnson [1] attributed the unusually higher amplitude of the Nc to the live paradigm used by Striano and colleagues. In the present study, the shape of the grand average demonstrates the effectiveness of our paradigm. We propose that very large, extended Nc can be observed in young infants when highly interesting social cues are employed.

One restriction of our findings is that we compared the infant's own name only with other first names. We cannot exclude the possibility that infants might show similar auditory ERP to objects after hearing other “special” words [21] such as “mommy” or deictic words such as “look” or “there!”. This must be subject for future studies, which might also benefit from the development of new techniques, such as simultaneous recording of EEG and eye tracking.

The present study provides new evidence that infants as young as 5 months benefit from “special” vocal cues when processing novel objects. Young infants not only detect their own name, but also use it to establish the relevance of information in the surrounding world. Future research will clarify the development of this skill and the way that various social cues interact to impact early social development and learning.

Since our findings are reliable across infants, as non-parametric statistics show, they also may have implications for understanding of early communicative disorders. Children with autism, for example, fail to respond to the own name in the first year of life [47], [48]. The current findings may thus lead to more sensitive diagnostic tool for such communicative disorders.

## Materials and Methods

### Participants

Thirty German infants (16 females, average age  = 149 days, SD = 6.90 days, range  = 137 to162 days) were included in the final sample. All infants were born full term (37–41 weeks) and in the normal birth weight range (>2500 g). An additional 25 infants were tested but excluded as a result of failing to reach the minimum requirements for adequate averaging of ERP data (n = 16), fussiness or crying (n = 6), experimenter error or technical problems (n = 3).

The minimum criterion for inclusion was at least 10 artifact-free trials in each of two conditions. For a discussion of this criterion see [49]. Ethical approval was obtained from the ethics committee of the Charité-Universitätsmedizin Berlin. Parents gave written informed consent for their children's participation in the study. Infants received a toy for participating.

### Stimuli and Procedure

Visual and auditory stimuli were presented. Auditory stimuli consisted of the infant's first name spoken in infant-direct-speech by a female voice. A large set of auditory files was prepared in advance, based on infants' names from the database of families who agreed to participate in infancy research. Names were taped with a DAT recorder, digitized at a 16-bit/44.1 kHz and presented via loudspeakers (mean SPL = 70 dB).

Visual stimuli consisted of 10 colorful photographs each showing a different object (an infant toy). All pictures were equated by luminance and low-level perceptual characteristics. Each object was shown on a white square, 224×201 pixels on average, resolving to 7.92×7.09 cm on a 90 Hz, 16-inch stimulus monitor. At the viewing distance of 70 cm, horizontal and vertical subtended visual angles were 6.47° and 5.80° respectively.

When a family was invited to participate, the lab assistant ensured the name stored in the auditory files database matched the infant's first name. She asked parents for the correct pronunciation and/or alternative nicknames of the infant. She also ensured that none of the control names used for that infant were used at home (e.g., father, mother or siblings' names).

Infants were presented with two equally probable stimuli: infant's own name vs. stranger's name. In order to control the influence of the variability of the control name (not always well controlled in previous works, see introduction), infants were randomly assigned to two groups. In one group they heard ten control names (five female and five male names), in the other group infants heard only one control name (matched by gender). Care was taken that for each infant all control names differed from infant's own name in the first phoneme. All control names were matched to the infant's own name by syllables number. All names were matched for loudness (mean SPL = 70 dB), but not for duration.

Infants sat on their mother's lap in a dimly lit, sound-attenuated and electrically-shielded cabin, at a viewing distance of 70 cm away from the stimulus monitor. Mothers were instructed to look straight ahead and not to influence the baby in any way. The experiment consisted of one block with 200 trials, 100 trials for each condition: infant's own name and stranger's name. It resulted in a 2×2 mixed design, with Group (ten control names vs. one control name) as a between-subjects factor and Name (infant's own name vs. stranger's name) as a within-subjects factor.

All stimuli were presented using the software ERTS (BeriSoft Corporation, Germany). Each experimental trial consisted of an auditory and a visual stimulus (see Figure 1). Triggers on the electroencephalogram (EEG) were time locked to the onset of both auditory and visual stimuli. This allowed the construction of two ERP averages for each participant. All trials started with a white cross on the screen centre and the simultaneous presentation of a name. The time window to present a name was fixed at 1500 ms (all presented names ranged from 396 to 989 ms, mean  = 650 ms; specifically for the infant's own name mean  = 648 ms and for strangers' names mean  = 651 ms; for details see Table 1). The name presentation was followed by a random interval between 750 and 850 ms, with the white cross still on the screen. The trial ended with the presentation of an object for 1000 ms. During the inter trials interval the screen was blank for a random period between 800 and 1200 ms. Experimental conditions were differentiated by the auditory portion of the trial. The presentation order was pseudo randomized with the constraint that no more than two identical conditions were presented in a row. In each block of 20 consecutive trials all objects were presented twice, once in each condition. An animated spiral and tone was presented when needed to reorient the infants' attention. If an infant became fussy, the experimenter gave the infant a short break. The session ended when the infant's attention could no longer be attracted to the screen. The behavior of the infants was video-recorded throughout the session for offline trial-by-trial editing of the EEG to ensure that the infant was looking at the screen for all included visual portion of the trials.

**Table 1:** Length of names.

Ten control names		One control name	
Infant's own name	Control names (average of 10 names)	Infant's own name	Control name
631	538	639	600
711	634	800	803
535	520	725	576
778	762	748	795
629	634	709	727
463	520	595	824
682	658	724	717
491	520	537	439
722	754	540	494
700	658	537	629
508	520	669	734
589	634	840	671
780	658	585	720
707	634	440	720
833	762	606	665

Length in ms of presented names for each infant for the two experimental groups. In the group with ten control names the average of the 10 control names is reported beside the length of the infant's own name. Note that the length of own name and control name does never perfectly match because names were matched by syllables' number only.

### Electrophysiological recordings

EEG was recorded continuously with Ag-AgCL electrodes from 23 scalp locations of the 10-20 system, referenced to the vertex (Cz). Data were amplified via a Twente Medical Systems 32-channel REFA amplifier (Twente Medical Systems International, Enschede, The Netherlands). Bipolar horizontal and vertical electro-oculargrams (EOGs) were recorded to control artifacts caused by eye movements. The electrical potential was digitalized at a 250 Hz sampling rate. A low-pass filter equal to .27 of the sampling rate ( = 67.5 Hz) was applied online during EEG acquisition. EEG was offline bandpass filtered (0.3–20 Hz, 1501 points) and re-referenced to the linked mastoids. The bandpass filter used in this study is the same used in a variety of visual ERP studies with infants across different labs. However, in auditory ERP studies with infants different filters are used, with their own advantages and disadvantages. For a detailed discussion see [50]. The filter has been applied as the first step of data editing on the continuous EEG to minimize data distortion. For analyses of auditory data with a different filter see Supplementary Information S1. For the elimination of artifacts caused by eye and body movements, EEG data were rejected offline whenever the standard deviation within a 200 ms gliding window exceeded 80 µV at EOG electrodes or 50 µV at any other electrode. Data were edited for artifacts by offline visual inspection as well. Auditory ERP included 200 ms baseline of blank screen from the inter trials interval and 1500 ms covering the entire name length; visual ERP included 200 ms baseline of white cross, 1000 ms of object presentation and 500 ms of inter trials interval. All the information present in the ERPs was analyzed. Components shaped as a peak (N200-600, Nc) were analyzed for both amplitude and latency of the peak. The other components not showing a clear peak (anterior positive shift, extended Nc) were analyzed as average amplitude, averaging all datapoints within the given time window.

Across conditions each infant contributed 20–79 trials (mean  = 32.57) to their auditory ERP average (10–38, mean  = 16.43 for infant's own name; 10–41, mean  = 16.13 for stranger's name; *t*(29) = .46, *P* = .65); and 20–80 trials (mean  = 34,60) to their visual ERP average (10–37, mean  = 17.00 for object preceded by infant's own name; 10–43, mean  = 17.60 for object preceded by stranger's name; *t*(29) = −1.02, *P* = .32). The two groups contributed an equal number of trials to both auditory (ten control names: means  = 31.13; one control name: means  = 34.00; *t*(28) = −.58, *P* = .57) and visual ERPs (ten control names: means  = 33.27; one control name: means  = 35.93; *t*(28) = −.50, *P* = .62).

## Supporting Information

Supplementary Information S1 Split-half analyses and different filter.
(0.04 MB DOC)
