# Supplementary material for: “Did You Call Me?” 5-Month-Old Infants Own Name Guides Their Attention
Source: PLoS One. 2010 Dec 3;5(12):e14208. doi: 10.1371/journal.pone.0014208 (PMC2997051; doi:10.1371/journal.pone.0014208)
Supplement: Supplementary Information S1 — Split-half analyses and different filter. (0.04 MB DOC) [file pone.0014208.s001.doc]

**Supplementary Information**

**Split-half analyses**

*ERPs to names*

To rule out the possibility that the repetition of one name might elicit fast learning that could interfere with the attentional mechanism we postulate, we have split the EEG to names into 2 portions: the first half of selected trials and the second half of selected trials. The splitting was done for each individual subject in both groups. This analysis allowed us to check for changes of the anterior positive shift and N200-600 component over the duration of the experimental session. According with a fast learning view, whether there was a learning process going on, the anterior positive shift should increase (or at least sustain) its amplitude over time in the group with one control name. We performed a 2 x 2 ANOVA with Group (ten vs. one control name) as between-subjects factor and Split-Half (first half vs. second half) as within-subjects factor. Both anterior positive shift and N200-600 were tested. For the anterior positive shift only an interaction of Group by Split-Half (*F*_(1,28)_ = 3.86, *P* = .06, η_p_^2^ = .121) was marginally significant, whereas no significances were found for the N200-600. Scheffé post hoc test on the anterior positive shift interaction did not reveal individual significant comparisons (all *P* > .3). Moreover, looking at the averages, in the one control name group the amplitude of the anterior positive shift decreases over time (first half: 5.60 µV; second half: 1.31 µV), and it does the opposite in the ten control names group (first half: 1.79 µV; second half: 3.84 µV). Although individual post hoc comparisons were not significant, this pattern is inconsistent with a fast learning hypothesis. If a learning process was going on in the one control name group, the potential should increase or at least be sustained over time. This pattern rather suggests that the infants in the one name group were probably more bored compared to those in the ten names group, which it is reasonable because the auditory stream in the one name group was constant and monotonous. If so, similar analyses for the time window 890-1000 ms in the ERPs to objects should show a decrease of potential.

*ERPs to objects*

We performed the same 2 x 2 ANOVA, with Group as between-subjects factor and Split-Half as within-subjects factor, on the time window 890-1000 ms. We found that in the first half of the accepted trials the amplitude was higher than in the second half of the accepted trials (*F*_(1,28)_ = 11.57, *P* < .003, η_p_^2^ = .292). This result is expected based on the prediction above and on the known characteristics of the Nc and the potentials that follow it. Moreover, although we did not find an interaction Group by Split-Half, looking at the averages the decrease of the potential over time is double in the group with one control name (first half: -12.74 µV; second half: -2.16 µV) compared to the group with ten control names (first half: -10.12 µV; second half: -5.55 µV). This result provides an explanation of why in the one control name group the extended Nc effect for the objects appears less pronounced than in the other group (see Figure 5 in the main article): the infants were probably bored and paid less attention. Interestingly, analyzing the two groups separately and considering only the left hemisphere (F3, FC3, C3) where the effect was more pronounced, in the ten names group the extended Nc was significantly higher to the objects preceded by the own name in the first half of the trials (*F*_(1,14)_ = 7.05, *P* < .019, η_p_^2^ = .335) and became not significant in the second half of the trials (*F*_(1,14)_ = 0.63, *P* < .441, η_p_^2^ = .043). In the group with one control name we observed the opposite: there was not significant difference in the first half of the trials (*F*_(1,14)_ = 0.14, *P* < .909, η_p_^2^ = .001) and a tendency turned out in the second half of the trials (*F*_(1,14)_ = 2.42, *P* < .142, η_p_^2^ = .147). This tendency became nearly significant (*F*_(1,14)_ = 3.91, *P* < .068, η_p_^2^ = .218) when considering only the subset of electrodes showing the effect in the group (F3, FC3). Notably, this approach to significance happened in a context of overall decreasing potential in the second half of the experimental session, when the participants’ attention was not optimal anymore. These findings can be interpreted as a facilitation in the ten names group due to the fact that the own name was presented 50% of the trials against 5% of each individual control name (infants were using statistical regularities in the speech stream to direct attention to the objects). But also a phonological facilitation is plausible. The infant’s own name “popped out” easier from the ongoing speech steam in the ten names group and infants were initially facilitated to detect the ostensive cue and to use it for object processing. Finally, it could also be that in the group with one control name the infants simply required longer time in order to understand that there was an ostensive signal directed to them, because the two sounds kept repeating at the same frequency and the control name acted as a confound in the artificial context of the experimental setting with no additional ostensive cues (such as direct eye contact) accompanying the own name.

**Different filter**

*ERPs to names*

Given that in auditory studies with infants a variety of offline filters are used, sometimes leading to different results (see [1]), here we repeated the main analyses on the ERPs to names with a bandpass filter .1-20 Hz.

For the anterior positive shift we found results overlapping those obtained in the main analysis: a main effect of Name (*F*_(1,28)_ = 5.83, *P* < .03, η_p_^2^ = .172) and an interaction of Group by Name (*F*_(1,28)_ = 5.12, *P* < .04, η_p_^2^ = .155). For the N200-600 component we found no significant main effects or interactions. The marginally significant interaction found in the main analysis was sensibly reduced by the new filter (*F*_(1,28)_ = 2.74, *P* = .11, η_p_^2^ = .089). These results suggest that the effects observed on the anterior positive shift are more stable than those observed on the N200-600 component. Based on the few available data [2,3] this is not very surprising, as positivities seem to precede negative ongoing waves in development.

**References**

1. Weber C, Hahne A, Friedrich M, Friederici AD (2004) Discrimination of word stress in early infant perception: electrophysiological evidence. Brain Res Cogn Brain Res 18: 149-161.

2. Sheehan EA, Mills DL (2008) The effect of early word learning on brain development. In: Friederici AD, Thierry G, editors. Early language development: bridging brain and behaviour. Amsterdam/Philadelphia: John Benjamins Publishing. pp. 161-190.

3. Maennel C, Friederici AD (2010) Prosody is the key: ERP studies on word segmentation in 6- and 12-month-old children. J Cognit Neurosci Supplement: 291.
